# Illustrated Manual of Operative Surgery and Surgical Anatomy

**Published:** 1852-09

**Authors:** 


Art. IV.—*Illustrated Manual of Operative Surgery and Surgical Anatomy.* By C. Bernard & C. Huette; edited, with notes and additions, by W. H. Van Buren, M. D., Surgeon to Bellevue Hospital; and C. E. Isaacs, M. D., Demonstrator of Anatomy in the College of Physicians and Surgeons, New York. H. Balliere: New York. 1852.

Such is the ample title of a work now in progress of publication in New York, under the superintendence of two distinguished physicians of that city, whose talents and learning eminently fit them for the enterprise. The original work was issued, a short time ago, at Paris, and has been received, every where, with great favor, on account of the extraordinary beauty and richness of its mechanical execution, which is far superior to that of any treatise on surgery that has ever appeared. Every surgical instrument, every surgical operation, and every part of surgical anatomy, "is most graphically and fully illustrated; thus enabling the merest tyro in the profession to comprehend their nature and object with as much facility almost as if they were spread out before him by the hand of a master. Indeed, it is impossible to conceive of any thing more perfect in its kind. The text has been well rendered by the American editors, who have spared neither labor nor expense to make the work worthy of the patronage of their countrymen. Here and there they have added a valuable note, elucidating some obscure or doubtful point in the original, or illustrating some operative procedure of some of our own surgeons. What greatly enhances the value of the work is the fact that all the plates are imported direct from Paris.

The *Manual* of Bernard & Huette is to be published in four parts, of which two are already before the public. We learn that one of the editors is now in Paris, to make arrangements for the speedy completion of the work. Mean while, we take much pleasure in recommending the parts already issued to the favorable consideration of our brethren, and in assuring them that they cannot obtain, anywhere, within the same compass, and for the same amount of money, so correct, beautiful, and graphic a guide to operative surgery. When completed, the work will find a place in the library of every educated physician in the country. G.

